# Intestinal Tuberculosis and Crohn’s Disease is Always a Diagnostic Challenge: A Case Report and Review of the Literature on the Importance of Fecal Mycobacterial Cultures and the Limitations of Latent Infection Testing

**DOI:** 10.7759/cureus.5689

**Published:** 2019-09-18

**Authors:** Adam Kurnick, Nir Bar, Nitsan Maharshak

**Affiliations:** 1 Sackler Faculty of Medicine, Tel Aviv University, Tel Aviv, ISR; 2 Department of Gastroenterology and Hepatology, Tel Aviv Sourasky Medical Center, Tel Aviv, ISR

**Keywords:** tuberculosis, crohn’s disease, latent tb infection, diagnosis, mycobacterium tuberculosis

## Abstract

Intestinal tuberculosis (TB) may mimic Crohn’s disease (CD) and may be overlooked where TB is not endemic. We present a case of an elderly patient with partial small bowel obstruction caused by intestinal TB, initially suspected to have ileal stricturing CD. In our case, the patient had multiple hospitalizations due to small bowel obstruction. She had a normal chest X-ray and a negative interferon-γ release assay (QuantiFERON Gold) done as screening prior to anti-tumor necrosis factor (TNF) therapy. Only the fecal mycobacterial culture was positive, which prevented the dismal outcome that immunosuppression would have on a patient with active TB.

We review the literature comparing the likenesses and dissimilarities between intestinal TB and CD. These include the disease epidemiology, clinical manifestations, imaging, endoscopy, histology, microbiology test sensitivities, and treatments. Intestinal TB is still in the differential diagnosis of CD, and no single test can exclude TB. It is important to remember fecal cultures are available to aid diagnosis when tissue is difficult to attain. Tests for latent TB infection (LTBI) are far from perfect, and clinical suspicion, along with imaging, endoscopic, and histologic findings, should always be integrated.

## Introduction

Intestinal tuberculosis (TB) was one of the main causes of small bowel obstruction before the 1960s until increased sanitation and anti-TB drugs reduced the incidence of mycobacterial disease [1]. Despite being considered rare in the past, a 2017 World Health Organization (WHO) report declared TB to be the ninth leading cause of death worldwide [2]. Immunodeficiency (mostly human immunodeficiency virus, HIV), increased immigration of people from countries that have a high incidence of TB, and the emergence of multidrug-resistant TB, have all significantly contributed to the increased incidences of TB in the Western world [3]. Intestinal TB shares many aspects with Crohn’s disease (CD) but is treated very differently.

## Case presentation

An 81-year-old female holocaust survivor of Jewish Ashkenazi descent presented to the hospital from a nursing home with recurrent vomiting of more than 20 times a day, diffuse abdominal pain, and bloody diarrhea (>10/day) without fever. Past medical history included chronic obstructive pulmonary disease, ischemic heart disease, diabetes, and hypertension. Prior surgical history included appendectomy due to acute appendicitis 11 years before admission. During the prior several years, she had recurrent admissions due to partial small bowel obstructions that manifested as vomiting and abdominal pain. Computed tomography (CT) scans revealed skip lesions of intestinal wall thickening, with a narrowing of the lumen and pre-stenotic dilation but no transition point. Two ileo-colonoscopies were endoscopically and histologically normal, though the lesion on the imaging could not be reached. The patient was referred to our gastrointestinal (GI) outpatient clinics but was lost to follow-up.

**Figure 1:**
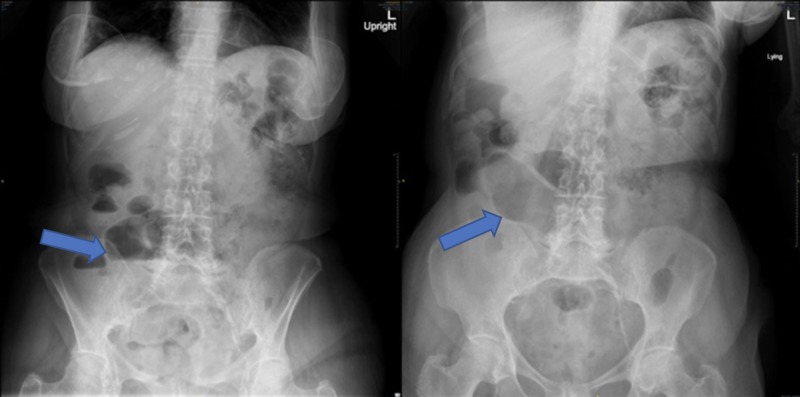
Left: Air-fluid levels (arrow). Right: non-specific loop dilation (arrow).

**Figure 2:**
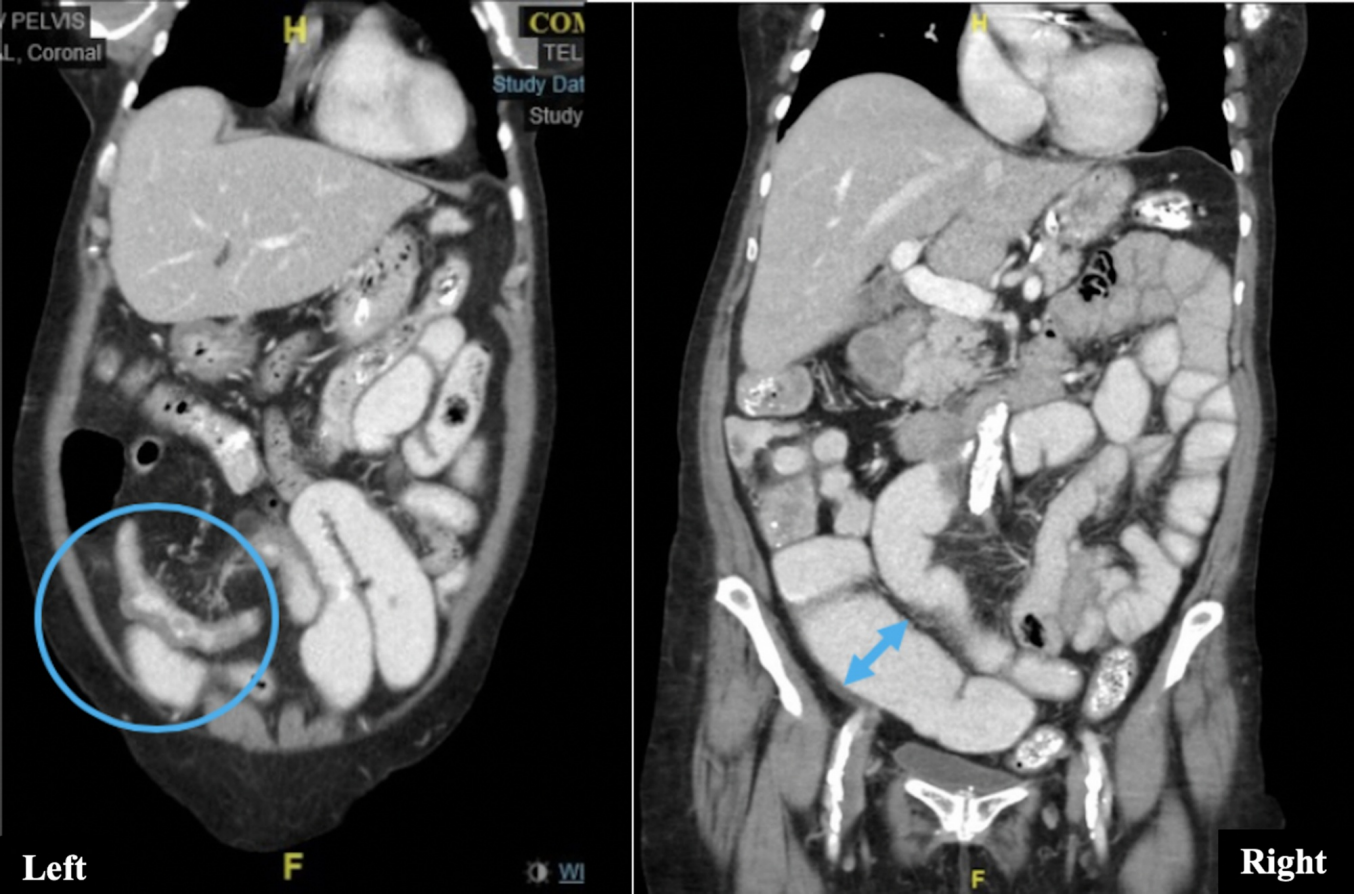
Left: Wall thickening of the distal ileum (circle). Right: Dilation of proximal bowel loops (double-headed arrow).

**Figure 3:**
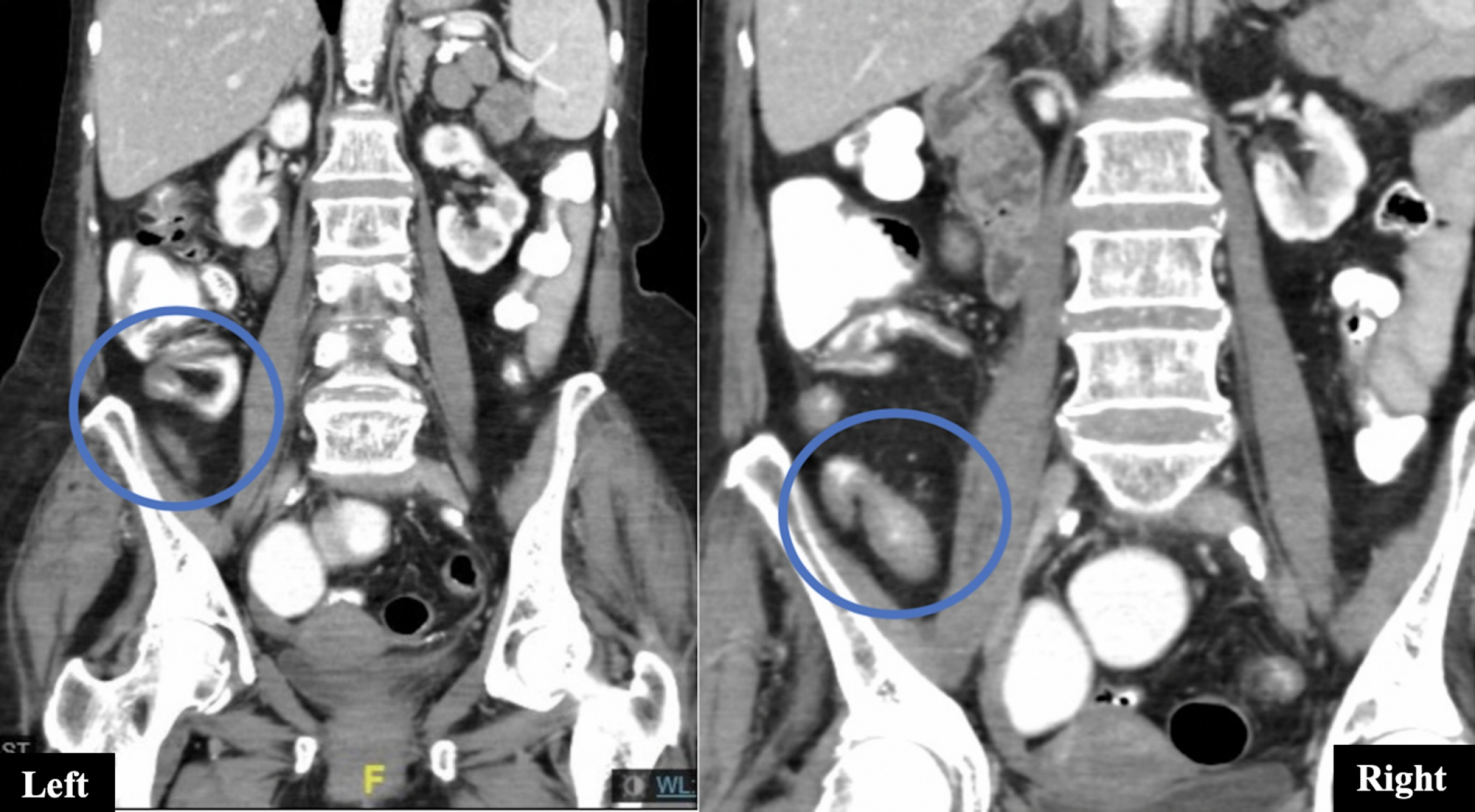
Left: CT scan on admission showing bowel wall thickening (circle). Right: CT scan post-corticosteroids therapy, showing worsening of wall thickening (circle; jejunal involvement is not shown). Computed tomography (CT)

On admission, respiratory and cardiovascular examinations were normal and abdominal exam revealed hyperactive bowel sounds and diffuse abdominal tenderness without peritoneal signs. Rectal examination was normal. Systemic lymphadenopathy was absent. Laboratory examination was normal except for hypokalemia (potassium = 3.0 meq/L) and slightly elevated C-reactive protein (CRP = 7 mg/L, normal values <5 mg/L). Chest X-ray was unremarkable and abdominal X-ray revealed distension of the loops and a few air-fluid levels with nonspecific dispersion (Figure 1). CT scans revealed increased wall thickness of the distal ileum and dilation of the proximal bowel loops, with oral contrast reaching the rectum (Figure 2). Esophagogastroduodenoscopy was unremarkable and the ileo-colonoscopy showed no significant endoscopic or histologic changes although the involved ileum was not reached. Fecal analysis for bacteria cultures, parasites, Clostridium difficile toxin, and acid-fast stains was negative. She was started on corticosteroid therapy for suspected Crohn’s disease (CD). Therapy resulted in an initial improvement of nausea and diarrhea. However, after a week, abdominal pain and vomiting recurred and additional abdominal CT imaging revealed a greater extent of ileitis and new proximal jejunal and duodenal involvement (Figure 3). A push enteroscopy was then performed, with a normal-appearing jejunum. A QuantiFERON Gold test was performed and returned negative prior to the initiation of anti-tumor necrosis factor (TNF) therapy for an apparent steroid-resistant CD. Luckily, at this point, fecal mycobacterial cultures had returned positive for Mycobacterium tuberculosis (TB).

Work-up for systemic TB involvement demonstrated positive gastric juice and negative sputum cultures, including a polymerase chain reaction (PCR) to TB. A lung CT scan revealed peribronchial thickening with tree-in-bud opacities. The standard TB treatment protocol was initiated and included isoniazid, rifampin, ethambutol, and pyrazinamide for two months, followed by four months of isoniazid and rifampin without any adverse events. Repeat stool cultures were negative for TB infection. Three months following the completion of treatment, the patient felt well, with no vomiting or diarrhea.

## Discussion

Intestinal TB and CD share clinical, radiographic, and histologic characteristics; thus, differentiation may pose a diagnostic challenge. No single test confirms CD and, occasionally, TB diagnosis and treatment should be assigned without microbiological confirmation, as described in a previous report [4]. The differing and similar aspects of the diseases are discussed below.

Epidemiology

An estimated 1.7 billion people worldwide were exposed to Mycobacterium tuberculosis, but only 5%-15% will develop an active TB infection. Risk factors for the development of TB infection include malnutrition, diabetes, smoking and alcohol consumption, and the likelihood of developing TB is much higher among those infected with HIV [2]. The majority of TB patients in Israel were found to be immigrants [5]. In a retrospective study of extrapulmonary TB in Israel between 1999 to 2010, there were roughly 1,000 reported cases of TB and the prevalence of extrapulmonary TB was shown to be increased with age [6]. On the contrary, CD incidence decreased with age [7]. It should be noted that in this case, our patient had a past history of being an immigrant, a Holocaust survivor, and a nursing home resident, which are all considered as risk factors for TB.

Clinical manifestations

Gastrointestinal (GI) mycobacterial spread can result from the ingestion of mycobacteria through contaminated food or sputum [3,8]. The involvement of the digestive system varies among patients. The area most commonly involved is the ileocecal region (42%), followed by the jejunum/ileum (35%), and colon (12%) [1]. Common symptoms are abdominal pain, weight loss, fever, vomiting, night sweats, gastrointestinal bleeding, bowel obstruction, and perforation [9-10], which may mimic fibro-stenotic or penetrating CD, which often involves the ileocecal or ileal region. Perianal disease is more common in patients with CD than in TB, but spiking fevers, as well as typical symptoms of pulmonary tuberculosis, are important clues for the latter. In our case, the patient's symptoms were non-contributory in differentiating between CD and TB, underlining the importance of epidemiologic risk factors and clinical suspicion.

Imaging

In CD, bowel walls are symmetrically thickened, as opposed to asymmetrical thickening in TB. Additionally, CD shows creeping fat, a hypervascular appearance of the mesentery, and the lymph nodes are small and homogeneous. In TB, the lymph nodes are large and necrotic, and ascites is sometimes present [8]. Our patient showed ileal and jejunal involvement, which is characteristic of intestinal TB. There was no evidence of creeping fat.

Endoscopy

The ulcers in CD are linear and aphthous with superficial ulcerations or cobblestoning of the colonic mucosa. Ulcers involving the rectum, sigmoid colon, descending and ascending colon, and jejunum occur significantly more in CD than in TB [11]. Hypertrophy of the mucosa can be observed in TB, as well as destruction or gaping of the ileocecal valve [3]. In a study comparing patients with proven intestinal TB to patients with CD, bloody stools, left colon involvement, and focally enhanced colitis on imaging were significantly more likely to have CD [11].

Histology

Granulomas are more commonly observed in intestinal TB as compared to CD. In TB, the granulomas are larger in size, lined by epithelioid histiocytes, and will be found caseating only in TB [11]. Since the involved intestine was inaccessible during her hospitalization (balloon-assisted endoscopy was not performed), neither endoscopic nor histologic parameters assisted in reaching our diagnosis.

Microbiology

Staining and culture of intestinal tissue, feces, or any type of fluid are a pivotal part of the TB diagnosis, but due to the slow growth of mycobacteria, it is often challenging to receive positive results. PCR also plays an important role in reaching a quick diagnosis [1]. In our case, only the fecal culture itself was positive. While not universally available to clinicians, we should remember its merit in microbial confirmation and sensitivity determination.

Test sensitivity

On its own, each test has limited sensitivity. Whether it is a smear, histology, or culture, sensitivity ranges from 13% to 70%. No single test can rule out TB. Combining all available data may increase sensitivity, and integrating it with imaging and endoscopic findings is usually necessary [1].

Treatment

When intestinal TB is diagnosed, standard first-line treatment includes four drugs: isoniazid, rifampin, pyrazinamide, and ethambutol. Surgical intervention is indicated in the case of failed medical treatment or in an unclear diagnosis [1,12]. In a study measuring responses to anti-TB treatment, 70% of patients had a complete resolution with medical treatment, and 8% required surgery due to non-response [13]. Our patient reported dramatic symptomatic improvement and did not require surgery.

The scope of this case report cannot encompass the treatment of CD, which often includes systemic immunosuppression that may exacerbate the mycobacterial infection.

Latent TB infection (LTBI)

Screening for LTBI is common practice before immunosuppressive treatment since it may cause the reactivation of LTBI. Tuberculin skin test (TST) (purified protein derivative (PPD)) and the interferon-γ release assays (IGRA) are used to detect such infections. However, many studies have shown these tests address LTBI and do not rule out active disease. Nash et al. found that approximately 25% of patients with active pulmonary TB were recorded as negative responders [14]. Additionally, an analysis of 10 studies measuring IGRA reactivity in patients with active TB found that approximately 21% of patients were negative responders [15]. In a meta-analysis of Asian studies that evaluated the accuracy of IGRAs in detecting active intestinal TB, IGRAs had 80% sensitivity and a negative predictive value of 87% [16]. Of course, the negative IGRA, theoretically allowing immunosuppression, could have resulted in catastrophic consequences in this frail, elderly patient if treated as a rule-out test.

## Conclusions

In conclusion, we present a case of intestinal TB mimicking CD. No single test is sensitive enough to exclude TB, and every effort should be made to pursue a microbial diagnosis. Every so often, we need to integrate epidemiologic, clinical, radiologic, endoscopic, and histologic factors to rule in TB and start treatment. Additionally, LTBI testing is associated with high false-negative rates for active disease and cannot be used to rule out an active TB infection. In our case, the availability of TB fecal cultures helped us reach the correct diagnosis and this option should be utilized when intestinal TB is suspected.
